# Online sexual harassment and negative mood in Croatian female adolescents

**DOI:** 10.1007/s00787-020-01506-7

**Published:** 2020-03-12

**Authors:** Kirstin Mitchell, Aleksandar Štulhofer

**Affiliations:** 1 MRC/CSO Social and Public Health Sciences Unit, University of Glasgow, Glasgow, UK; 2 Department of Sociology, Faculty of Humanities and Social Sciences, University of Zagreb, 10000 Zagreb, Croatia

**Keywords:** Online sexual harassment, Negative mood, Resilience, Female adolescents, Repeated measurement

## Abstract

Online sexual harassment (OSH) appears to be a relatively frequent phenomenon, particularly for older adolescents. It is also a gendered experience. Compared to their male peers, female adolescents are more likely to experience OSH and find it upsetting. This study sought to explore the role of resilience in explaining the association between online sexual harassment (OSH) and negative mood (i.e., depression and anxiety symptoms) among female adolescents. Using data from a panel sample of 477 female Croatian adolescents (age at baseline = 15.8 years; SD = 0.48) and two-wave cross-lagged path analysis, we investigated OSH, changes in depression/anxiety symptoms, association between OSH and negative mood, and the role of resilience. During the 26-month period under observation, OSH and negative mood were associated cross-sectionally, but not longitudinally. This suggests the negative mood effects of OSH exposure may be short-lived or that factors other than OSH explain changes in negative mood over time. Resilience was consistently and negatively associated with negative mood, but not OSH. In adolescent girls with low levels of resilience, OSH was associated with negative mood; no such relationship was observed among their highly resilient peers. Experiences other than OSH appear to be more pertinent in predicting symptoms of negative mood in older adolescent girls over time. Given that resilience attenuated the relationship between OSH and negative mood, efforts to increase resilience to online challenges may be more helpful than efforts to limit or control young people’s online exposure.

## Introduction

Sexual harassment is an age-old problem, but rapidly changing digital technology has brought new manifestations and complex challenges. Defined as unwanted sexual attention [1], sexual harassment can occur in internet chat rooms and social networking sites, as well as via sending of explicit images, videos or text messages. Sexual harassment is gendered: most studies find that young women are more likely to experience it and young men to perpetrate it. In addition, among those exposed to sexual harassment, young women are more likely than men to find it upsetting [2–7]. It should be noted, however, that some studies have found little or no gender differences [8] as well as that the moderating effect of gender on the link between sexual harassment and emotional adjustment may not be consistent [5].

Regular internet use is nearly universal among young people [7] and increasingly shapes both their understanding of sexual norms and expressions of sexuality [9]. Estimates of online sexual harassment (OSH) vary significantly, but most studies suggest it is not uncommon, particularly for older adolescents. In the Youth Internet Safety Survey in US [7], OSH in the last year was reported three times as often by young women (12%) compared with young men (4%). The survey also documented a significant increase in experience of online harassment in general (from 6% in last year in 2000 to 11% in 2010), primarily reflecting that young people increasingly interact online with their peers [7]. A European internet study of online safety [6] found that 15% of 11–16 year olds had received ‘sexual messages or images of people naked or having sex’ from their peers in the last year, with one quarter of these young people finding this experience fairly or very upsetting. It is worth bearing in mind that sexual interactions between young people on the internet are frequent and only a small proportion of these interactions are unwanted, exploitative or distressing [9].

Several studies find associations between sexual harassment and cyberbullying on the one hand, and symptoms of depression and anxiety on the other [3, 10], though causal direction is not always certain. Experience of sexual harassment is predictive of emotional distress, problematic substance use and subsequent victimization [2], while depression may fuel risk-taking behavior online such as high disclosure [11], which may draw unwanted attention. Difficulty with ‘offline’ relationships may also lead young people to seek friendship online in ways that make them vulnerable to harassment and/or victimization [12]. Due to a dearth of longitudinal studies in this field, mechanisms underlying links between OSH and psychological health and well-being are poorly understood [13].

Resilience, defined as the ability to ‘overcome the negative effects of risk exposure’ [14] is a significant predictor of mental health in adolescents [15]. Resilience is strongly implicated in positive adjustment following early traumatic sexual experiences [16] and has been suggested as a protective factor that moderates the link between risk exposure and negative outcomes, including sexual risk taking [14]. However, the role of resilience in the association between exposure to sexual harassment and internalizing problems has not been addressed. Whether resilience may reduce the likelihood of further exposure is currently also unknown.

### Current study

In an era of heightened concerns about adolescent exposure to OSH, we sought to address a gap in understanding possible association between OSH and negative mood in the period between middle and late adolescence, and the role of resilience. The following two research questions were explored: (RQ1) does baseline frequency of OSH predict negative mood levels 2 years later and (RQ2) does resilience buffer the association between the two constructs? Following the literature and previous analyses that used the same dataset [17], we focused on female adolescents because of their higher risk of exposure [5], greater likelihood of distress [3], and higher vulnerability to negative mood [18].

## Method

### Participants

A panel sample of high-school sophomores, aged 15.8 (SD = 0.48) years at baseline, was recruited from 14 secondary schools in Rijeka, the third largest city in Croatia,^1^ as a part of a longitudinal study of the role of sexually explicit material in adolescent sexual socialization and sexual health (for more details about the study see [17]). For logistical reasons, only larger schools (with 63% of the city’s second-grade high-school student population) were selected in the sample. The initial survey (*n* = 1287) took place in December 2015 (Time 1) and was repeated five times in 5–6-month intervals. The final wave (T6; *n* = 892) was carried out in March 2018. The notable reduction in panel size was mostly due to the fact that students who enrolled in a 3-year vocational school program completed their education and left school before T5. For the current study, we only used data from female students who participated in both T1 and T6 (*n* = 477).

Attrition rate reflected school absenteeism (due to illness, educational field trips, obligatory out-of-school practical courses, and truancy) as well as occasional difficulties in questionnaire linking (approx. 1.4 questionnaires per class per wave). We carried out multivariate logistic regression analysis to investigate possible bias introduced by attrition. Independent variables were age, mother’s and father’s education, academic achievement, resilience, and reported frequency of OSH and negative mood symptoms at baseline. Two significant differences emerged between female students who participated in both T1 and T6 (coded 1) and those who did not (coded 0). Compared to the latter, the former group was characterized by higher odds of reporting better grades (AOR = 1.70, *p* = 0.000) and somewhat more educated mothers (AOR = 1.36, *p* = 0.035).

### Procedure

Data were collected by a self-administered paper and pencil survey administered in classrooms. To maximize confidentiality, large screens were placed between participants. In addition to being printed on the first questionnaire page, information required for students’ consent was also delivered by a research assistant. Contact information for a youth psychological health center was provided at the end of the questionnaire, which took about 20–25 min to complete. Prior to the study launch, all parents/carers were sent a leaflet with basic information about the study. All study procedures were approved by the Ethical Research Committee of the Faculty of Humanities and Social Sciences, University of Zagreb.

### Measures

Online sexual harassment (OSH) was measured at baseline and the final wave by asking participants whether they were ever (T1) or in the past 6 months (T6) sent an unwanted (1) text message with sexual content, (2) unwanted sexually explicit photograph of the sender or (3) unwanted X-rated photograph clip. A 5-point scale (1 = never, 2 = once, 3 = 2–3 times, 4 = 4–5 times, 5 = 6 times or more) was used to anchor answers. The composite indicator, the values of which ranged from 3 to 15, had acceptable internal consistency at each wave (Cronbach’s *α* was 0.82 at T1 and 0.89 at T6).

Symptoms of depression and anxiety were also assessed at T1 and T6 with the Patient Health Questionnaire for Depression and Anxiety (PHQ-4 [19]), a brief 4-item measure which was used to assess symptoms of depression (two items, e.g., Little interest or pleasure in doing things) and anxiety (two items, e.g., Feeling nervous, anxious or on edge) experienced in the 2 weeks preceding the survey. A 4-point scale ranging from 1 = not at all to 4 = nearly every day was used to record the frequency of symptoms. The construct was characterized by good reliability (Cronbach’s *α* was 0.86 at both T1 and T6), and had acceptable stability over time (*r* = 0.43).

Resilience was measured at baseline, T4, T5, and T6. It was assessed by a shortened version of the Connor-Davidson Resilience Scale [20]. This brief 5-item measure (e.g., I can deal with whatever comes, I can stay focused under pressure, and I think of myself as strong person) was pre-tested in a preparatory pilot study carried out in early 2015. A 5-point Likert-type scale, ranging from 1 = it never relates to me to 5 = it always relates to me, was employed to record answers. Following the notion that resilience is not a static individual trait [14], but a dynamic process [21], a composite variable was built by averaging the five items across data collection waves. Over time, the indicator had satisfactory reliability (Cronbach’s *α* ranged from 0.75 at baseline to 0.79 at T6) and stability (*r* = 0.73–0.53). To reflect middle to late adolescents’ resilience, the four composites were then averaged into a single one. For multi-group testing, this global resilience indicator was categorized (i.e., tertilized) into three roughly equally sized groups: (1) low resilience, (2) moderate resilience, and (3) high resilience.

### Analytical strategy

**Fig. 1:**
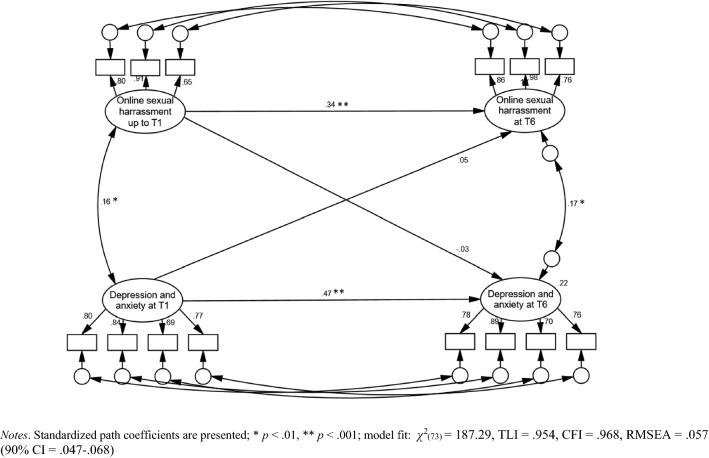
Path Analytic Assessment of Associations between online sexual harassment (OSH) and negative mood in female Croatian adolescents over 26 months (*n* = 477)

Cross-lagged path analysis was used to explore associations between OSH and negative mood over the period of 26 months. The analytical procedure consisted of several steps. As the precondition for exploring structural associations, the key constructs’ measurement models were tested for configural, weak factorial, and strong factorial invariance across time by applying progressively more restrictive equality constraints [22]. The standard test of Chi-square difference (*Δχ*^2^) was used for comparisons of nested models. After confirming strong factorial invariance of depression and anxiety symptoms and partial strong factorial invariance of OSH, we explored the two-wave cross-lagged path analytic model presented in Fig. 1. Next, the model was controlled for participants’ resilience at baseline; the composite indicator was added to the model and connected to all other latent constructs (OSH at T1 and T6 and negative mood at T1 and T6). Finally, as recommended by Fergus and Zimmerman [14], a multi-group analysis was employed to address the protective role of resilience by directly comparing paths between OSH and negative mood among female adolescents characterized by low, moderate, and high resilience. Model fit was evaluated using *χ*^2^, CFI, and RMSEA statistics. Following standard guidelines for longitudinal structural equation assessment, TLI and CFI values ≥ 0.95 and RMSEA values ≤ 0.05 were taken to represent excellent fit [22]. Due to multivariate non-normality (the distribution of OSH was particularly skewed), path analytic models were bootstrapped with 5000 resamples to produce 95% confidence intervals around the relevant estimates [23].

Considering that < 2% of information was missing on the key variables, full information maximum likelihood (FIML) estimation was used to deal with missing data [24]. Data nestedness in school classes was disregarded based on the finding that only 3% of variance in female adolescents’ OSH and < 7% of variance in negative mood was explained at the level of class.

All statistical analyses were carried out using AMOS 24 and IBM SPSS 24 software packages.

## Results

### Sample characteristics, OSH, and negative mood at baseline

The majority of participants (80.3%) reported living with both parents at baseline. The majority of participants reported that their mother and/or father were secondary educated (56.9% and 60.5%, respectively). A college-educated parent was reported by over a third of participants. A substantial minority (28.9%) of students reported attending religious services at least once a month (16.2% reported no religiosity). At baseline, 18.0% of students were sexually active.

Over a third of participants (37.5%) reported (lifetime) OSH at baseline. At T6, OSH in the past 6 months was reported by 15.7% female adolescents. Overall, 42.6% of participants experienced OSH at some point. On average, exposure to OSH in this panel sample was low at both data collection points (*M*_T1_ = 4.33, *SD* = 2.37 and *M*_T6_ = 3.54, *SD* = 1.75). Importantly, the average levels of depression and anxiety symptoms decreased between T1 (*M* = 9.51, *SD* = 3.37) and T6 (*M* = 8.52, *SD* = 2.98).

### RQ1: relationship between OSH and negative mood over time

Table 1 shows bivariate associations between the key constructs. Of the four possible associations between OSH and negative mood, only the one between the two constructs at T6 was statistically significant, albeit small (*r* = 0.18). There were no associations between adolescent resilience and OSH, but resilience was consistently and negatively related to depression and anxiety symptoms reporting (*r* = − 0.25 and *r* = − 0.26). The effects were small to moderate in size.

**Table 1:** Associations between the key constructs

	2	3	4	5	*M* (SD)
(1) OSH at T1	0.30**	0.13**	0.04	− 0.03	4.33 (2.37)
(2) OSH at T6		0.09	0.18**	0.01	3.54 (1.75)
(3) D/A at T1			0.43**	− 0.25**	9.51 (3.37)
(4) D/A at T6				− 0.26**	8.52 (2.98)
(5) Resilience					17.85 (3.53)

*OSH* online sexual harassment, *D/A* depression and anxiety; *p < 0.05, **p < 0.01

A multivariate, path analytic assessment of links between OSH and negative mood is shown in Fig. 1. In the model, which was characterized by adequate fit to the data (*χ*^*2*^(73) = 187.29, TLI = 0.954, CFI = 0.968, RMSEA = 0.057 [90% CI 0.047–0.068]), the key constructs were significantly associated at both baseline (*r* = 0.16, *p* = 0.003) and T6 (*r* = 0.17, *p* = 0.001). However, cross-lagged paths were not significant, indicating that baseline levels of OSH were not predictive of reported depression and anxiety symptoms 2 years later and vice versa. Bootstrapping the model with 5000 resamples confirmed this pattern of (non) significant structural links.

### RQ2: the role of resilience

With resilience controlled for in the path analytic model (*χ*^2^(83) = 206.79, TLI = 0.951, CFI = 0.966, RMSEA = 0.056 [90% CI 0.046–0.066]), the associations between T1 and T6 levels of OSH and negative mood remain significant and almost identical in size. Corroborating bivariate findings, adolescents’ resilience was significantly related to depression and anxiety symptoms at T1 (*β* = − 0.30, *p* = 0.000) and T6 (*β* = − 0.15, *p* = 0.004), but not to OSH.

To address the question of whether resilience can buffer the association between the key constructs, we carried out a multi-group path analytic assessment, in which groups represented participants with different resilience levels (Table 2). At bivariate level, the three groups differed significantly in the reported OSH at T6 (*F* = 3.38, *p* = 0.035) and depression and anxiety symptoms at both T1 (*F* = 14.78, *p* = 0.000) and T6 (*F* = 28.68, *p* = 0.000), with higher levels of OSH and negative mood observed in the low compared to the high resilience group. The multi-group model, which was characterized by acceptable fit (*χ*^2^(108) = 481.24, TLI = 0.903, CFI = 0.914, RMSEA = 0.052 [90% CI 0.045–0.059]), pointed to substantial differences in the associations between OSH and negative mood among the three groups. In the low resilience group, associations between OSH and negative mood were significant both at T1 (*r* = 0.22, *p* = 0.034) and T6 (*r* = 0.24, *p* = 0.027)—unlike in the high resilience group, where neither reached statistical significance. The moderately resilient participants were characterized by a non-significant association at T1 and a significant relation at T6 (*r* = 0.32, *p* = 0.003).

**Table 2:** Structural associations between OSH and negative mood in three resilience groups

	Low resilience group (*N* = 116)	Moderate resilience group (*N* = 119)	High resilience group (*N* = 107)
	*r* (SE) *p*	*r* (SE) *p*	*r* (SE) *p*
OSH T1 ↔ negative mood T1^a^	0.22 (0.06) 0.034	− 0.05 (0.05) 0.583	0.09 (0.08) 0.363
OSH T6 ↔ negative mood T6^a^	0.24 (0.05) 0.027	0.32 (0.03) 0.003	0.08 (0.02) 0.511
	*β* (SE) *p*	*β* (SE) *p*	*β* (SE) *p*
OSH T1 → negative mood T6^b^	− 0.03 (0.08) 0.760	0.02 (0.08) 0.811	− 0.11 (0.06) 0.300
Negative mood T1 → OSH T6^b^	− 0.04 (0.12) 0.675	0.08 (0.06) 0.418	0.07 (0.05) 0.436

^a^Cross-sectional associations

^b^Longitudinal associations

Bootstrapping the multi-group analysis, as a remedy for multivariate non-normality, produced the same pattern of differences between the three resilience groups, with one exception: in the low resilience group, the association between the key constructs at T6 failed to reach significance (*p* = 0.109). All other findings appeared robust.

## Discussion

To address a gap in understanding the mental health impact of OSH, we sought to explore the relationship between OSH and negative mood in a panel sample of female Croatian adolescents. Over a 26-month period covering the transition between middle and late adolescence, we observed a decrease in reported depression and anxiety levels. Although OSH and negative mood were associated cross-sectionally, at both study waves, baseline OSH levels were unrelated to subsequent change in negative mood and vice versa. Reported resilience buffered the association between the two constructs, so that the significant links characterized female adolescents with low, but not high resilience levels.

The lifetime prevalence of OSH in the current study (43%) appears substantially lower than a recent study among 17-year-old female Californian adolescents (68%) [25]. Differences in measurement of OSH (the operationalization of cyber sexual harassment in the Reed et al. study included being pressured to sext) are likely to explain some of the disparity. Other explanations include cultural differences in willingness to report and in what is deemed harassment as well as the possibility of actual differences in exposure.

Given the absence of longitudinal links between the two constructs, the cross-sectional associations between OSH and negative mood at T1 and T6—which remained significant after controlling for participants’ resilience—suggest either that the negative impact of OSH is relatively short-lived or that the relationship is spurious, or confounded by other factors. One of these may be parental engagement and support during earlier developmental phases. A number of cross-sectional studies have concluded that parental engagement protects, either directly or indirectly (i.e., through fostering self-esteem and competence [26]), against OSH [5], negative mood [27, 28], and unwanted sexual experiences [7, 9]. Other candidate explanations are that striving for peer popularity and/or peer conformism intensifies emotional reactions to OSH, or that being a part of a peer group characterized by deviant behaviors, increases the likelihood of being exposed to OSH. Future research should assess the role of these intra- and inter-personal characteristics in the dynamics of adolescent OSH and negative mood.

The protective role of resilience, enabled by a combination of personal assets (such as self-control and agency [21]) and external resources (mostly related to parental engagement [14]), has been recognized in different domains of adolescents’ life. The current study’s finding that high levels of resilience buffered the link between female adolescents’ OSH and negative mood is compatible with such observations. The findings that higher levels of resilience were consistently related to lower frequency of negative mood symptoms at baseline [15], but not to OSH, are in line with the conceptualization of resilience as limiting negative affective reaction to problematic experiences and outcomes. The lack of systematic association with OSH makes intuitive sense, given that resilience implies good coping strategies in response to negative online experiences [29], but cannot prevent externally initiated exposure. Given this, the fact that female adolescents who reported low resilience experienced more OSH at T6 compared to their highly resilient peers is intriguing. It suggests that teens characterized by higher resilience may actually be better at avoiding OSH. This is perhaps because of their ability to identify and avoid online situations that might place them at higher risk of exposure. It might also be due to differences in online networks and friendship groups, such that highly resilient young women are members of friendship groups with more supportive norms around online behaviors [30].

### Study strengths, limitations, and future directions

The current study has several strengths, including repeated measurements over a developmentally important 26-month period and the use of robust and reasonably powered analytical approach. In addition, there was no evidence of relevant attrition bias beyond educational attainment. However, there are also several limitations. Apart from self-reported nature of our data, it should be noted that the measure of OSH differed in its timeframe; at baseline we asked for lifetime experience, while at T6 we inquired about the past 6 months. Our study also did not distinguish between distressing and non-distressing OSH, although the measure did focus on ‘unwanted’ experiences, which the literature specifies as problematic. Future studies should address this issue and how it may reflect on the role of resilience. Finally, compared to lengthier indicators, the brief measure of depression/anxiety symptoms used in the current study is characterized by a more limited variability. Although this may have affected the estimation of key associations, the marginal effect sizes observed in the current study suggest that the limitation is minor.

Future research could also explore a few other plausible moderators of the observed cross-sectional association between OSH and negative mood. Of particular relevance might be peer support, which can be assessed in detail using ego-centered network analysis [31], illicit drug and alcohol use [25], and the parallel experience of (cyber)bullying [32]—which may amplify emotional reactions to OSH.

## Conclusions

In a world of rapidly developing digital technologies and evolving social norms about their use, young people need support in establishing healthy norms of communication and patterns of use. The observed non-ignorable levels of OSH experienced before the age of 16 years suggest that school-based interventions to build internet literacy skills and support should be in place by early adolescence. These could include specific skills (such as blocking senders, reporting, recognizing ‘safe’ and ‘unsafe’ chat rooms, etc.) but should also address the potential mental health sequelae of unwanted exposure by creating an enabling environment for young people to talk about their experiences of OSH and receive support.

Many parents who feel anxious about their children’s risk of online exposure seek to monitor internet use [4, 29, 33] but these efforts become less feasible as their children move into middle and late adolescence. Furthermore, digital media also provides important opportunities for young people and to limit risk can also imply limiting opportunity [34]. Perhaps, a more productive strategy for parents would be to assist their children in building resilience to challenges posed by new communication technologies.

## Footnotes

1 Historically a port town with close ties to Italy, Rijeka lost most of its industry during the process of post-socialist transition in the 1990s. During the past three decades, the municipality of Rijeka has been uninterruptedly governed by social democrats—in contrast to most other parts of the country, in which more conservative Christian democrats have been dominating. In 2013, the county of Rijeka was one of the two counties (of the total of 21) that rejected a proposal to amend the constitutional definition of marriage as a heterosexual union.
